# Effect of a low‐fat diet on serum triglyceride and cholesterol concentrations and lipoprotein profiles in Miniature Schnauzers with hypertriglyceridemia

**DOI:** 10.1111/jvim.15880

**Published:** 2020-10-06

**Authors:** Panagiotis G. Xenoulis, Paul J. Cammarata, Rosemary L. Walzem, Jan S. Suchodolski, Jörg M. Steiner

**Affiliations:** ^1^ Gastrointestinal Laboratory, Department of Small Animal Clinical Sciences, College of Veterinary Medicine and Biomedical Sciences Texas A&M University College Station Texas USA; ^2^ Laboratory for Cardiovascular Chemistry, Department of Chemistry Texas A&M University College Station Texas USA; ^3^ Department of Poultry Science, and Faculty of Nutrition Texas A&M University College Station Texas USA; ^4^ Present address: The Dow Chemical Company Freeport TX USA

**Keywords:** hypercholesterolemia, hypertriglyceridemia, nutrition, nutritional treatment, treatment

## Abstract

**Background:**

Hypertriglyceridemia is common in Miniature Schnauzer (MS). Dietary management of hypertriglyceridemia is important, but no studies are available.

**Hypothesis/Objectives:**

To evaluate the effect of a commercially available low‐fat diet on serum triglyceride and cholesterol concentrations and lipoprotein profiles in MS with hypertriglyceridemia.

**Animals:**

Sixteen MS with hypertriglyceridemia and 28 MS without hypertriglyceridemia.

**Methods:**

Prospective clinical trial. Four blood samples (1‐2 months before and 1 day before diet change and 2 and 3 months after the dogs were fed the low‐fat diet) were collected from the MS with hypertriglyceridemia.

**Results:**

Serum triglyceride concentrations for the 2 samples after the diet change (median of sample 3 = 177 mg/dL; range, 48‐498; median of sample 4 = 168 mg/dL; range, 77‐745) were significantly lower than the 2 samples before the diet change (median of sample 1 = 480 mg/dL; range, 181‐1320; median of sample 2 = 493 mg/dL; range, 114‐1395; *P* < .001). Serum cholesterol concentrations for the 2 samples after the diet change (mean for sample 3 = 257 mg/dL, SD = 82.2; mean for sample 4 = 178 mg/dL, SD = 87.4) were also significantly lower than the 2 samples before the diet change (mean for sample 1 = 381 mg/dL, SD = 146.1; mean for sample 2 = 380 mg/dL, SD = 134.7; *P* < .001). Before the diet change, 15/16 (94%) of hyperlipidemic MS were classified as hyperlipidemic based on their lipoprotein profiles alone. After the diet change, significantly fewer MS (7/16; 44%; odds ratio = 19.3; 95% CI = 2.0‐184.0; *P* = .006) were classified as hyperlipidemic based on lipoprotein profile analysis.

**Conclusions and Clinical Importance:**

The study diet was effective in reducing serum triglyceride and cholesterol concentrations and correcting lipoprotein profiles in MS with hypertriglyceridemia.

**Abbreviations**
BCS: body condition score
cTSH: canine thyroid‐stimulating hormone
HDL: high‐density lipoproteins
LDA: linear discriminant analysis
LDL: low‐density lipoproteins
MS: Miniature Schnauzer
OR: odds ratio
SIR: sliced inverse regression
Spec cPL: specific canine pancreatic lipase
T4: thyroxin
TRL: triglyceride‐rich lipoproteins
VLDL: very low‐density lipoproteins

## 1 INTRODUCTION

Idiopathic or primary hyperlipidemias in dogs are a group of metabolic disorders of diverse etiology that are more common in certain breeds.^1^ The phenotypic appearance of these disorders as determined by routine clinicopathological testing is quite uniform and typically characterized by increased serum concentrations of triglycerides, cholesterol, or both. However, more in‐depth analysis of these lipid abnormalities, by lipoprotein profiling, often reveals important differences among breeds.^2^

Primary hypertriglyceridemia was first described in the Miniature Schnauzer (MS) and occurs in dogs of this breed in the United States and several other countries.^3^, ^4^, ^5^ Hypertriglyceridemia is present in 33% of 192 MS from the United States investigated, with >75% of dogs over 9 years of age being affected, suggesting that it is possibly the most common lipid abnormality in dogs.^4^ Hypertriglyceridemia in MS is typically characterized by increases of various degrees in very low density lipoproteins (VLDL) and chylomicrons, with or without concurrent hypercholesterolemia.^2^, ^6^

Several pieces of evidence suggest that hypertriglyceridemia in MS is a condition of major clinical importance. While many animals are asymptomatic,^4^ hypertriglyceridemia in this breed has been linked to other disorders such as hepatobiliary disease (eg, gall‐bladder mucocele, vacuolar hepatopathy),^7^, ^8^ pancreatitis,^9^, ^10^ insulin resistance,^11^ glomerular disease (eg, glomerular lipidosis),^12^, ^13^ ocular disease (eg, ocular lipid deposits, lipemic uveitis),^14^, ^15^ and neurological abnormalities (eg, seizures).^1^

We have recently reported a detailed analysis of the lipoprotein profiles of MS with hypertriglyceridemia using a novel lipoprotein density profiling method.^2^ However, the biochemical, metabolic, and genetic bases of hypertriglyceridemia in MS are yet to be elucidated. Therefore, definitive recommendations for effective prevention and specific management of this condition are currently unavailable. Due to the serious and potentially even fatal diseases that have been associated with hypertriglyceridemia in MS, management of hypertriglyceridemia is mandatory even in dogs in which clinical signs are not present.^1^ The most commonly recommended initial approach in the management of hypertriglyceridemia is the use of a low‐fat diet.^1^ There are several commercially available diets marketed as “low‐fat,” although their actual lipid content might vary substantially between one another. To our knowledge, none of these diets have been evaluated with regard to their efficacy in the management of hypertriglyceridemia in MS.

The hypothesis of our study was that a commercially available low‐fat diet would be effective in improving hypertriglyceridemia and lipoprotein profiles in hyperlipidemic MS. The aim of the study was to evaluate the effect of a commercially available low‐fat diet on serum triglyceride and cholesterol concentrations and lipoprotein profiles in MS with hypertriglyceridemia.

## 2 MATERIALS AND METHODS

### 2.1 Dogs

#### 2.1.1 Group 1

Miniature Schnauzers with hypertriglyceridemia of various degrees were included in this group. These dogs were selected from a pool of >300 MS that were enrolled as part of several ongoing projects related to hypertriglyceridemia in this breed. The requirements for inclusion of dogs in group 1 in the present study were: (a) the presence of hypertriglyceridemia (dogs with combined hypertriglyceridemia and hypercholesterolemia were also included in the study if all other requirements were met; dogs with hypercholesterolemia alone were excluded); (b) consuming diets that were not labeled as “low‐fat”; (c) absence of any clinical signs at the time of initial blood collection; (d) no history of diseases or current use of drugs known to affect lipid metabolism^1^; (e) a body condition score (BCS), between 4 and 6 on a scale of 1 to 9 (the BCS was assessed by the referring veterinarian using a printed guide with pictures and instructions that was provided to each referring veterinarian); and (f) willingness of each dog's owners to enroll their dog in the study.

Diagnostic testing included a CBC, serum biochemistry profile, urinalysis, thyroid profile (ie, total T4, canine thyroid‐stimulating hormone [cTSH], free T4 by equilibrium dialysis), and specific canine pancreatic lipase concentration (Spec cPL) measurement. This testing was performed to evaluate dogs for the possibility of being hyperlipidemic secondary to other conditions such as hypothyroidism or diabetes mellitus. No function testing for hyperadrenocorticism was performed; however, consistent clinical signs for this condition were not identified. Based on the historical information for each dog and the results of the tests performed, secondary causes of hypertriglyceridemia were excluded with reasonable certainty and, therefore, all hypertriglyceridemic dogs enrolled in our study were presumed to have primary idiopathic hypertriglyceridemia.

#### 2.1.2 Group 2

A group of healthy MS without hypertriglyceridemia served to provide normative values for the lipoprotein profile portion of the study. The lipoprotein profiles of dogs in group 2 were used to assess whether lipoprotein profiles of hypertriglyceridemic MS approached those of healthy MS after the diet change. This approach was used because typical reference intervals are not available for MS lipoprotein profiles. The requirements for inclusion of dogs in group 2 were: (a) to have serum triglyceride and cholesterol concentrations within the respective reference intervals; (b) consuming diets that were not labeled as “low‐fat”; (c) absence of any clinical signs at the time of initial blood collection; (d) have no history of diseases or current use of drugs known to affect lipid metabolism; (e) have a BCS between 4 and 6 on a scale of 1 to 9); and (f) willingness of each dog's owners to enroll their dog in the study.

### 2.2 Study design

Each 1 of the dogs in group 1 had a total of 4 blood samples collected. The first sample (sample 1) was used to investigate the presence of hypertriglyceridemia and select which dogs would be candidates for enrollment into the study. Dogs with hypertriglyceridemia that also met all the criteria for enrollment mentioned above were chosen to continue with the study. In order to confirm the results of the initial sample and to investigate the variability of hypertriglyceridemia over time, a second blood sample (sample 2) was collected 1 to 2 months after the collection of the initial blood sample. During that time, the owners were instructed to not make any changes to the diet type or amount, or the activity of their dogs. If hypertriglyceridemia was confirmed with the second sample, the dogs were placed on the study diet (see below). Owners were instructed to gradually switch their dogs from the original diet to the study diet over 7 days. Because all dogs had a normal BCS, the amount of diet was determined based on the normal weight guide on the package of the diet. In addition, the owners were instructed to weigh their dogs once a week and adjust the amount of food accordingly in order to maintain a relatively stable body weight for the duration of the study. Additional instructions given to the owners involved ensuring that the study diet was fed exclusively to the dogs enrolled in the study, with the exception of small quantities of steamed vegetables (carrots and broccoli) that could be used as treats. No lipid‐lowering medications were used in any of the dogs for the duration of the study.

Approximately 7 to 9 weeks after the dogs had been exclusively on the study diet, a third blood sample (sample 3) was collected. Finally, a fourth sample (sample 4) was collected approximately 2 to 4 weeks after the third sample, and while the dogs were still exclusively on the study diet. This was done in order to confirm the results of the 3rd sample and to investigate the variability of serum triglyceride and cholesterol concentrations over time while the dogs were on the low‐fat diet.

Serum triglyceride and cholesterol concentrations were measured in all samples and compared between time‐points. Two cut‐off points were used for categorical analysis of serum triglyceride concentrations before and after diet change. The first cut‐off point was the upper limit of the reference interval of the assay (108 mg/dL; see below). However, increases in serum triglyceride concentrations that are slightly above the reference interval are considered mild and might not be associated with any appreciable risk for complications. In addition, there is anecdotal evidence of age‐related increases in serum triglyceride and cholesterol concentrations in dogs other than Miniature Schnauzers. Therefore, a second cut‐off point (500 mg/dL) was selected to indicate dogs that are considered to have severe hypertriglyceridemia and possessed of an appreciable risk for complications. The value of 500 mg/dL was selected as a value commonly used in clinical practice as indicative of extreme hypertriglyceridemia; there is no evidence‐based cut‐off for serum triglyceride concentrations that indicates an increased risk for all conditions associated with hypertriglyceridemia. Lipoprotein profiles were determined as previously described^2^ and comparisons made between time‐points (samples 1‐4) and between groups (hyperlipidemic vs. nonhyperlipidemic).

Group 2 dogs were enrolled on a 1‐time basis and no follow‐up samples were collected from these dogs. As mentioned above, these dogs were only used to provide normative lipoprotein profile data for apparently healthy MS. Serum triglyceride, cholesterol, and Spec cPL concentrations were also measured in all samples of dogs in this group but were not used for any comparison with group 1 (see above).

### 2.3 Study diet

The diet selected for the present study was a commercially available therapeutic diet labeled as “low‐fat” in dry form (Royal Canin Gastrointestinal Low‐fat, Royal Canin USA, Inc, St. Charles, Missouri). The fat content of the study diet was 18.6 g of fat per 1000 Kcal.

### 2.4 Blood collection and handling

Owners that agreed to participate in the study were each sent a styrofoam box containing ice packs and the material necessary for blood collection and were asked to schedule an appointment with their veterinarian for the blood collection. All dogs were required to be fasted for a minimum of 12 hours before each scheduled blood collection. Ten milliliters of blood were collected from each dog into a red‐top tube (with no additive). Blood samples were centrifuged immediately after clot formation and the serum separated from the clot. Serum samples were sent to the Gastrointestinal Laboratory packed on ice by overnight courier. Serum samples were stored at −80°C until further use.

### 2.5 Standardized questionnaire

Owners and/or primary care veterinarians of all dogs were asked to complete a standardized questionnaire for each dog and for each time a blood sample was collected. Questions covered date of birth, sex, body weight, BCS, current diet(s), current medications, and current and past health history of the dogs. Questionnaires from all dogs were reviewed to determine whether the dogs fit the inclusion criteria for each group and whether any of the variables evaluated changed during the study (eg, body weight or BCS).

### 2.6 Ethics approval

The owners of each dog enrolled in the study had to sign an informed owner consent form. The study protocol was reviewed and approved by the Clinical Research Review Committee at Texas A&M University (TAMU‐CRRC# 2008‐37).

### 2.7 Assays

Serum triglyceride (reference interval = 26‐108 mg/dL) and cholesterol (reference interval = 124‐335 mg/dL) concentrations as well as serum biochemistry profiles were determined by use of analytically validated automated enzymatic assays (Roche/Hitachi MODULAR ANALYTICS D 2400 module, Roche Diagnostics, Indianapolis, Indiana). Serum Spec cPL concentration (reference interval = ≤200 μg/L) was measured using an analytically validated immunoassay as described elsewhere.^15^ Serum total T4 concentration was measured by a solid‐phase chemiluminescent competitive assay (Immulite 2000 Canine Total T4, Siemens Healthcare Diagnostics, Deerfield, Illinois). Serum free T4 concentration was measured using a commercial equilibrium dialysis radioimmunoassay (Free T4 [by ED], Antech Diagnostics, Irvine, California). Serum cTSH concentration was measured by a solid‐phase, 2‐site chemiluminescent immunometric assay (Immulite 2000 Canine TSH, Diagnostic Products Corporation, Los Angeles, California).

### 2.8 Lipoprotein profile analysis

Lipoprotein profiling was carried out using a bismuth sodium ethylenediaminetetraacetic acid (NaBiEDTA) density gradient ultracentrifugation method as previously described.^2^, ^16^ The sodium salt of BiEDTA has been described as a novel solute forming a self‐generating density gradient during ultracentrifugation of serum samples for the separation of lipoproteins.^16^Briefly, for each sample, 1284 μL of a 0.18 M NaBiEDTA (Bismuth Sodium Ethylenediaminetetraacetate, TCI AMERICA, Portland, Oregon**)** gradient solution was added into a 1.5 mL tube. The fluorescent probe 6‐((N‐[7‐nitrobenz‐2‐oxa‐1,3‐diazol‐4‐yl]amino)hexanoyl)sphingosine (NBD C_6_‐ceramide, Molecular Probes, Inc, Eugene, Oregon) was reconstituted with 1 g/mL dimethyl sulfoxide to form a 1 mg/mL solution, and 10 μL of this solution were added to each tube to label the lipoproteins. Finally, 6 μL of serum was added to each tube. The mixture was vortexed at 1400 rpm for 10 seconds and 1150 μL of the mixture transferred into an ultracentrifuge tube (1 mL, 11 × 34 mmThickwall, Polycarbonate, Beckman Coulter, Inc, Brea, California). The mixture was incubated for 30 minutes at 5°C in order to saturate lipoproteins with the fluorescent probe. The solution was then centrifuged at 120 000 rpm (513 000*g*), at 5°C, for 6 hours in a Beckman Optima ultracentrifuge (TLX‐110, Beckman Coulter Optima TLX‐120 Ultracentrifuge, Beckman Coulter, Inc) with a 30° fixed angle TLA 120.2 rotor (TLA**‐**120.2 rotor, Beckman Coulter, Inc). A quality control sample was included in each run to verify that proper operating conditions were achieved. Immediately after ultracentrifugation, the top of each sample was carefully layered with 250 μL of hexane and imaged without delay.

Separated lipoproteins were imaged using a custom‐built fluorescence imaging system consisting of a digital camera (Digital Microfire Camera, Optronics, Goleta, California) with a MH‐100 metal halide continuous light source (MH‐100, Dolan‐Jenner Industries, Boxborough, Massachusetts) located in a dark room. Two filters (SCHOTT North America, Inc, Elmsford, New York) matching the excitation (blue‐violet filter centered at 407 nm) and emission (a yellow emission long pass filter with a cut‐on wavelength of 515 nm) characteristics of NBD C_6_‐cermide were used. A gain of 1.0000, a target intensity of 30%, and an exposure time of 53.3 ms were selected. In order to be analyzed, the image of each tube following ultracentrifugation was converted to a density profile using a commercially available software program (Origin 7.0, Microcal Software, Inc, Northampton, Massachusetts). A tube coordinate scale was established where 0.0 mm is the top of the tube and 34.0 mm is the bottom of the tube.^2^, ^16^ The average intensity was then plotted as a function of the tube coordinate.

The above described methodology identifies 11 distinct density lipoprotein fractions in dogs based solely on density characteristics.^2^ Because the functional characteristics and composition of most lipoprotein density subfractions in dogs are currently unknown, all density subfractions can only be nominally assigned to traditional functional classes such as low‐density lipoproteins (LDL) or high‐density lipoproteins (HDL). Density subfraction data are reported as previously described density ranges identified as R1 to R11 where: R1 (*d* < 1.017 g/mL), R2 (*d* = 1.019‐1.023 g/mL), R3 (*d* = 1.023‐1.029 g/mL), R4 (*d* = 1.029‐1.039 g/mL), R5 (*d* = 1.039‐1.050 g/mL), R6 (*d* = 1.050‐1.063 g/mL), R7 (*d* = 1.063‐1.091 g/mL), R8 (*d* = 1.091‐1.110 g/mL), R9 (*d* = 1.110‐1.133 g/mL), R10 (*d* = 1.133‐1.156 g/mL), and R11 (*d* = 1.156‐1.179 g/mL).^2^ Based on a previously published classification, and based solely on their density characteristics, these fractions could be classified as: triglyceride‐rich lipoproteins (TRL; chylomicrons and VLDL; *d* < 1.017 g/mL), LDL_1_ (*d* = 1.019‐1.023 g/mL), LDL_2_ (*d* = 1.023‐1.029 g/mL), LDL_3_ (*d* = 1.029‐1.039 g/mL), LDL_4_ (*d* = 1.039‐1.050 g/mL), LDL_5_ (*d* = 1.050‐1.063 g/mL), HDL_2b_ (*d* = 1.063‐1.091 g/mL), HDL_2a_ (*d* = 1.091‐1.110 g/mL), HDL_3a_ (*d* = 1.110‐1.133 g/mL), HDL_3b_ (*d* = 1.133‐1.156 g/mL), and HDL_3c_ (*d* = 1.156‐1.179 g/mL), respectively.^2^ The abovementioned previously reported classification scheme for lipoprotein fractions was used in our study.^2^

### 2.9 Statistical analysis

Commercial statistical software packages were used for all statistical analyses (SPSS 16.0, SPSS, Inc, Chicago, Illinois; Prism5, GraphPad, San Diego, California; R, http://www.r-project.org/). Data were analyzed for normal distribution using the Shapiro‐Wilk test. Summary statistics for continuous variables are reported as means (±SD) for parametric data and medians and ranges for nonparametric data. Categorical data are presented as counts (n) and percentages.

Normally distributed data were analyzed using *t* tests, while non‐normally distributed data were analyzed using Mann‐Whitney tests. For multiple comparisons, repeated measures 1‐way analysis of variance (ANOVA) was used for normally distributed paired data followed by Bonferroni analysis, while the Friedman's test was used for non‐normally distributed paired data followed by Dunn's post hoc multiple comparison tests. Proportions were compared between groups using Fisher's exact tests with calculation of the odds ratio (OR) and the 95% confidence interval (95% CI) to test the possibility of an association between categorical variables. Sliced inverse regression (SIR) or logistic regression analysis was used to test whether there was a relationship between group (diet, time‐point) and lipoprotein profiles. The SIR provides a linear discriminant analysis (LDA) value that ranks individuals within the group and generates graphical plots to show separation in groups. Significance was set at *P* < .05 for all analyses.

## 3 RESULTS

### 3.1 Dogs

A total of 39 dogs with hypertriglyceridemia had owners willing to approve their participation in the study and were initially considered for enrollment into group 1. Twelve of these dogs were then excluded from the study because they were diagnosed with conditions that are known or suspected to affect lipid metabolism (ie, hypothyroidism, pancreatitis, pregnancy). The owners of another 11 dogs submitted the initial 2 samples needed for the study (before the diet change) but decided to not proceed with the study at that time. These owners were concerned about their dogs' hypertriglyceridemia and decided to proceed with the use of lipid‐lowering medications. Thus, 16 dogs completed the study. The characteristics for all dogs included in the study are shown in Tables 1 and 2. The 16 dogs that completed the study had a median BCS of 5 (range, 4‐6) and a median age of 8.5 years (range, 6.7‐11.9 years). Eleven dogs were female (all spayed) and 5 dogs were male (all castrated).

**TABLE 1:** Characteristics of all Miniature Schnauzers included in the study (n−44)

Group characteristics	Hyperlipidemic	Normolipidemic	*P* value
Total number, n	16	28	—
*Patients characteristics*			
Age (y), median (range)	8.5 (6.7‐11.9)	9.1 (7.1‐12.2)	.57
Sex, male/female (neutered)	5 (5)/11 (11)	14 (8)/14 (5)	.34
Body weight (kg), median (range)	8.36 (5‐10.88)	7.37 (5.44‐10.7)	**.03**
BCS, median	5	5	
BCS category, n (%)			.13
≤5	14 (87.5%)	28 (100%)	
>5	2 (12.5%)	0 (0%)	
*Clinicopathologic variables (RI), median (range)*			
Serum triglyceride (26‐108 mg/dL)	480 (181‐1320)	54 (19‐108)	**<.001**
Serum cholesterol (124‐335 mg/dL)	362.6 (158‐575)	168.4 (121.7‐316.1)	**<.001**
Serum total T4 (1.61‐3.6 μg/dL)	2.3 (1.2‐5.05)	1.785 (0.5‐5.28)	.52
Serum cTSH (≤0.6 ng/mL)	0.235 (0.09‐1.24)	0.203 (0.029‐0.86)	.74
Serum free T4 (0.7‐3.1 ng/dL)	1.35 (0.6‐4.0)	1.5 (0.4‐2.5)	.62
Serum Spec cPL (0‐200 μg/L)	93 (29‐473)	29 (12‐386)	.32
Serum glucose (60‐120 mg/dL)	104 (79‐131)	98 (51‐125)	1

*Note*: Bold face values indicate statistical significance at *P* < .05.

Abbreviations: BCS, body condition score (range of possible scores: 1‐9); cTSH, canine thyroid‐stimulating hormone; RI, reference interval; Spec cPL, specific canine pancreatic lipase.

**TABLE 2:** Characteristics of all hyperlipidemic Miniature Schnauzers in the study (n = 14). Initial serum cholesterol concentrations were unknown for 2 of the 16 dogs. Therefore, these dogs are not included in this table

Group characteristics	HTGL alone	HGTL + HCHOL	*P* value
Total number, n	6	8	—
*Patients characteristics*			
Age (y), median (range)	9.7 (8.6‐10.9)	7.7 (6.7‐11.9)	.13
Sex, male / female	1/5	4/4	.3
Body weight (kg), median (range)	7.27 (5‐8.39)	9.15 (8.26‐10.88)	**.003**
BCS, median	5	4.5	
BCS category, n (%)			.43
≤5	5 (83.3%)	8 (100%)	
>5	1 (16.7%)	0 (0%)	
*Clinicopathologic variables (RI), median (range)*			
Serum triglyceride (26‐108 mg/dL)	271.5 (218‐1215)	559 (181‐1320)	1
Serum cholesterol (124‐335 mg/dL)	251.7 (158‐296)	511 (359‐575)	**<.001**
Serum total T4 (1.61‐3.6 μg/dL)	2.35 (1.12‐2.55)	2.5 (1.56‐5.05)	.58
Serum cTSH (≤0.6 ng/mL)	0.16 (0.09‐0.47)	0.31 (0.15‐0.88)	.63
Serum free T4 (0.7‐3.1 ng/dL)	1.2 (0.6‐2.2)	2.4 (0.7‐4)	1
Serum Spec cPL (0‐200 μg/L)	137 (31‐473)	85.5 (29‐449)	.54
Serum glucose (60‐120 mg/dL)	103 (79‐112)	105.5 (89‐131)	.47

*Note*: Bold face values indicate statistical significance at *P* < .05.

Abbreviations: BCS, body condition score (range of possible scores: 1‐9); cTSH, canine thyroid‐stimulating hormone; HCHOL, hypercholesterolemia; HTGL, hypertriglyceridemia; RI, reference interval; Spec cPL, specific canine pancreatic lipase.

Data on the diets the dogs were on before the diet change were collected. However, these data were impossible to analyze either because the dogs were on several different commercial diets at the same time or because they were being fed a combination of commercial diets and home‐made diets. As mentioned above, none of the commercial diets were labeled as “low‐fat’ and the owners were instructed to not change the amount or type(s) of diet or the dog's activity and lifestyle for the duration of the trial (with the exception of the study diet).

Group 2 consisted of 28 Miniature Schnauzers that had a median BCS of 5 (range, 4‐6) and a median age of 9.1 years (range, 7.1‐12.2 years). Fourteen dogs were female (8 spayed) and 14 dogs were male (5 castrated).

### 3.2 Serum triglyceride and cholesterol concentrations in group 1

**FIGURE 1:**
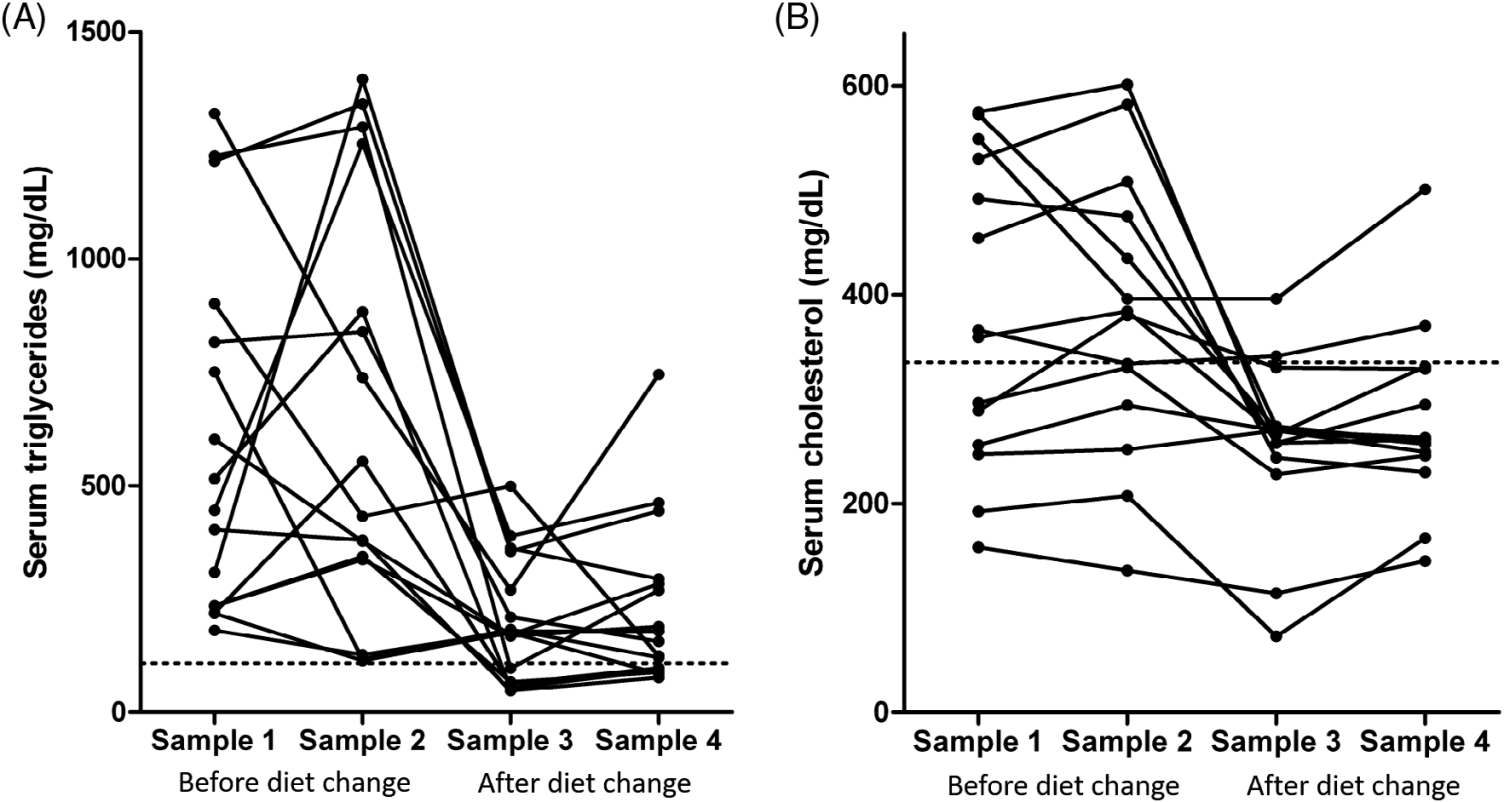
A, Serum triglyceride and, B, cholesterol concentrations in dogs with hypertriglyceridemia before and after diet change. Samples 1 and 2 were collected while dogs were on their original diets. Samples 3 and 4 were collected approximately 8 and 12 weeks, respectively, after the dogs were placed on the study diet. There was a significant decrease in serum triglyceride (*P* < .001) and cholesterol (*P* < .001) concentrations after the diet change. The dotted lines represent the upper limit of the reference interval. See also Tables 4 and 5

Analysis using the Friedman's test (repeated measures nonparametric 1‐way ANOVA) showed that serum triglyceride concentrations were significantly lower after the diet change (median for sample 3 = 177 mg/dL; range, 48‐498; median for sample 4 = 168 mg/dL; range, 77‐745) than before (median for sample 1 = 480 mg/dL; range, 181‐1320; median for sample 2 = 493 mg/dL; range, 114‐1395; *P* < .001; Figure 1A; Table 3). Tukey's post hoc test indicated that there were no significant differences between samples 1 and 2 (ie, while dogs were on their original diets) or between sample 3 and 4 (ie, while dogs were on the study diet). There were significant differences between samples before and after the diet change (*P* < .05 for all; Table 4). Furthermore, significantly more dogs had hypertriglyceridemia in group 1 before (16/16) than after the diet change (11/16; OR = 15.8; 95% CI = 0.8‐314.8; *P* = .04). Low‐fat diet consumption also significantly reduced the number of dogs with serum triglyceride concentrations >500 mg/dL in group 1 from 8/16 to 1/16; (OR = 33.0; 95% CI = 1.7‐643.6; *P* = .002) after the diet change.

**TABLE 3:** Serum triglyceride and cholesterol concentrations in Miniature Schnauzers with idiopathic hypertriglyceridemia (n = 16) before (samples 1 and 2) and after (samples 3 and 4) dietary intervention

Variable	Before low‐fat diet				After low‐fat diet				
	Sample 1		Sample 2		Sample 3		Sample 4		
	Median (range)	n (%) values above RI	Median (range)	n (%) values above RI	Median (range)	n (%) values above RI	Median (range)	n (%) values above RI	Difference/association
Triglycerides	480 (181–1320)	16/16 (100%)	492.5 (114–1395)	16/16 (100%)	177 (48‐389)	11/16 (68.75%)	167.5 (77–745)	11/16 (68.75%)	***P* < .001**
Cholesterol	362.6 (158–575)	8/14 (57.14%)	357.05 (136‐601)	8/14 (50%)	262 (73‐396)	3/16 (18.75%)	258 (122‐501)	2/16 (12.5%)	***P* = .003**

*Note*: P values in bold indicate significant difference or association at *P* < .05.

Abbreviation: RI, reference interval.

**TABLE 4:** *P* values for multiple comparison testing (Tukey post hoc test) for serum triglyceride concentrations in dogs with hyperlipidemia before (sample 1 and sample 2) and after (sample 3 and sample 4) low‐fat diet consumption

Sample 1 vs sample 2: *P* = .96
**Sample 1 vs sample 3: *P* = .004**
**Sample 1 vs sample 4: *P* = .002**
**Sample 2 vs sample 3: *P* = .004**
**Sample 2 vs sample 4: *P* = .003**
Sample 3 vs sample 4: *P* = .93

Serum cholesterol concentrations were available for all 4 time points for 14 of the 16 dogs in group 1. Repeated measures 1‐way ANOVA analysis showed that serum cholesterol concentrations after low‐fat diet consumption (mean of sample 3 = 257 mg/dL; SD = 82.2; mean for sample 4 = 278 mg/dL; SD = 87.4) were significantly lower than when dogs consumed their usual diet (mean of sample 1 = 381 mg/dL; SD = 146.1; mean for sample 2 = 380 mg/dL; SD = 134.7; *P* < .001; Figure 1B; Table 3). Post‐ANOVA analysis using Bonferroni's correction for multiple comparisons indicated that there were significant differences between individual comparisons before and after low‐fat diet consumption, with the exception of samples 2 and 4, which approached but did not reach statistical significance (Figure 1B; Tables 3 and 5). However, there was no significant difference in the proportion of dogs that had hypercholesterolemia before (8/16) and after the diet change (3/16; *P* = .14).

**TABLE 5:** *P* values for multiple comparison testing (Tukey post hoc test) for serum cholesterol concentration in dogs with hyperlipidemia before (sample 1 and sample 2) and after (sample 3 and sample 4) diet change

Sample 1 vs sample 2: *P* = 1
**Sample 1 vs sample 3: *P* = .01**
**Sample 1 vs sample 4: *P* = .05**
**Sample 2 vs sample 3: *P* = .01**
Sample 2 vs sample 4: *P* = .07
Sample 3 vs sample 4: *P* = .23

### 3.3 Lipoprotein profile analysis

**FIGURE 2:**
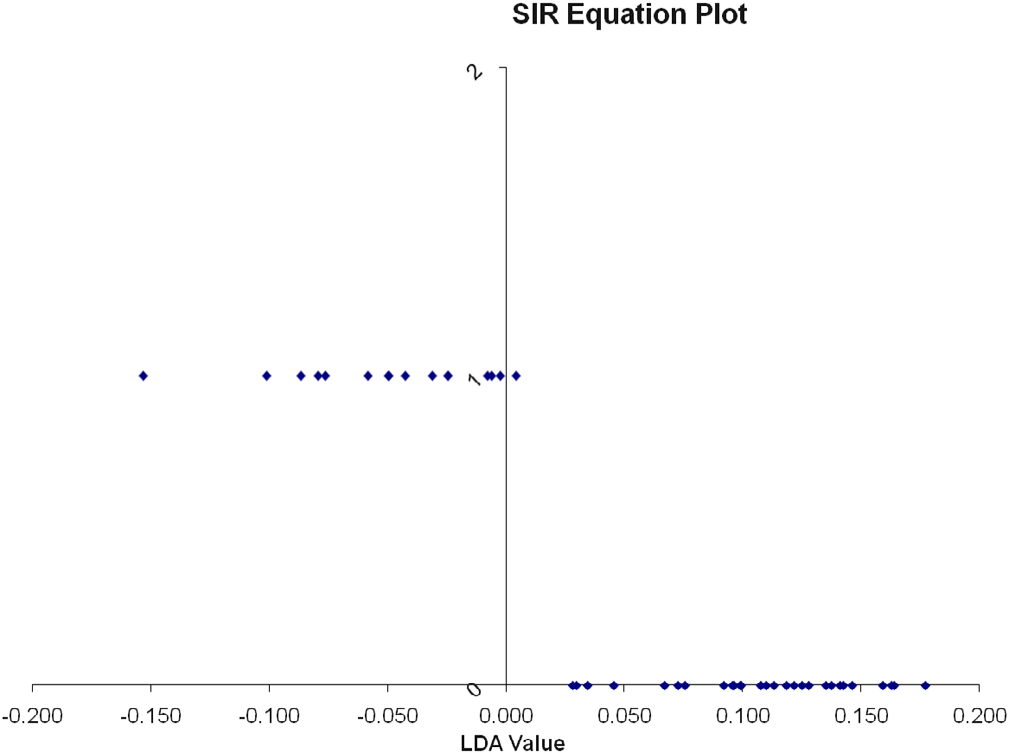
One dimensional SIR plot showing classification of dogs into groups based on lipoprotein density distribution before the diet change. The vertical line represents the line that separates the 2 groups based on lipoprotein density distribution. The LDA value provides a ranking value for each dog. The dogs represented by the dots that are at the bottom of the graph are the dogs of the control group. Their lipoprotein profiles plot them all to the right of the vertical line. The dogs represented by the dots at the top of the graph are the dogs of group 1 (with hypertriglyceridemia) before the diet change. They are all classified as a separate group from the control dogs with the exception of 1 dog that is classified as borderline normal. LDA, linear discriminant analysis; SIR, sliced inverse regression

**FIGURE 3:**
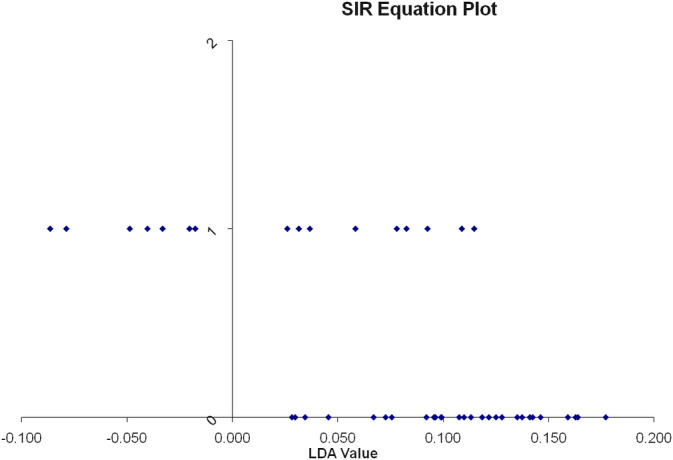
One dimensional SIR plot showing classification of dogs into groups based on lipoprotein profile analysis after the diet change. The vertical line represents the line that separates the 2 groups based on their lipoprotein profile analysis. The LDA value provides a ranking value for each dog. The dogs represented by the dots that are at the bottom of the graph are the dogs of the control group. Their lipoprotein profiles plot them all to the right of the vertical line. The dogs represented by the dots at the top of the graph are the dogs of group 1 (with hypertriglyceridemia) after the diet change. Nine of the 16 dogs are now classified to the right of the vertical line together with the control dogs, suggesting that they are considered normal based on their lipoprotein profile analysis. The remaining 7 dogs are classified separately from the control dogs even after the diet change. Note that there is a clear separation between dogs of group 1 that responded to the diet change and those that did not. LDA, linear discriminant analysis

Efficacy of the low‐fat diet intervention was also assessed by whether or not lipoprotein density subfractions of dogs in group 1 became more similar to those of normotriglyceridemic, Group 2, dogs following low‐fat diet consumption. Before the diet intervention, there was a 94% separation between group 1 and group 2 dogs using SIR analysis (Eigenvalues = 0.7748; *P* < .001; Figure 2). One Miniature Schnauzer from group 1 with a serum triglyceride concentration of 181 mg/dL was misclassified by SIR as a control dog. Therefore, 15/16 (94%) of hyperlipidemic MS were classified as hyperlipidemic based on their lipoprotein profiles alone. Following low‐fat diet consumption significantly fewer MS (7/16; 44%; OR = 19.3; 95% CI = 2.0‐184.0; *P* = .006) were classified as hyperlipidemic based on lipoprotein profile analysis. Lipoprotein density subfractions of the majority of the dogs in group 1 (56%) were classified as normal following low‐fat diet consumption for an average of 12 weeks (Figure 3). Removing the intact dogs in the control group from the analysis (to exclude the possibility that their intact status could have affected the results) did not change the results. Thus, the low‐fat diet intervention was effective in relieving lipoprotein abnormalities in 56% of hyperlipidemic dogs within 12 weeks.

Logistic regression analysis of the lipoprotein profiles of group 1 dogs consuming their usual diet showed that dogs that eventually responded to low‐fat diet feeding could be separated with 88% accuracy from dogs that did not respond to the low‐fat diet before the low‐fat diet intervention. The logistic regression model fit the data well (Chi‐square = 8.99; *P* = .003; −2 log likelihood = 4.09; Nagelkerke *R* ^2^ = 0.9; Hosmer‐Lemeshow test *P* = 1.0). The distinguishing features of lipoprotein density profile of nonresponders include lower LDL fractions (mainly LDL_1_ and LDL_2_) and higher HDL fractions (mainly HDL_2a_, HDL_3b_, and HDL_3c_) than responders (Table 2).

**FIGURE 4:**
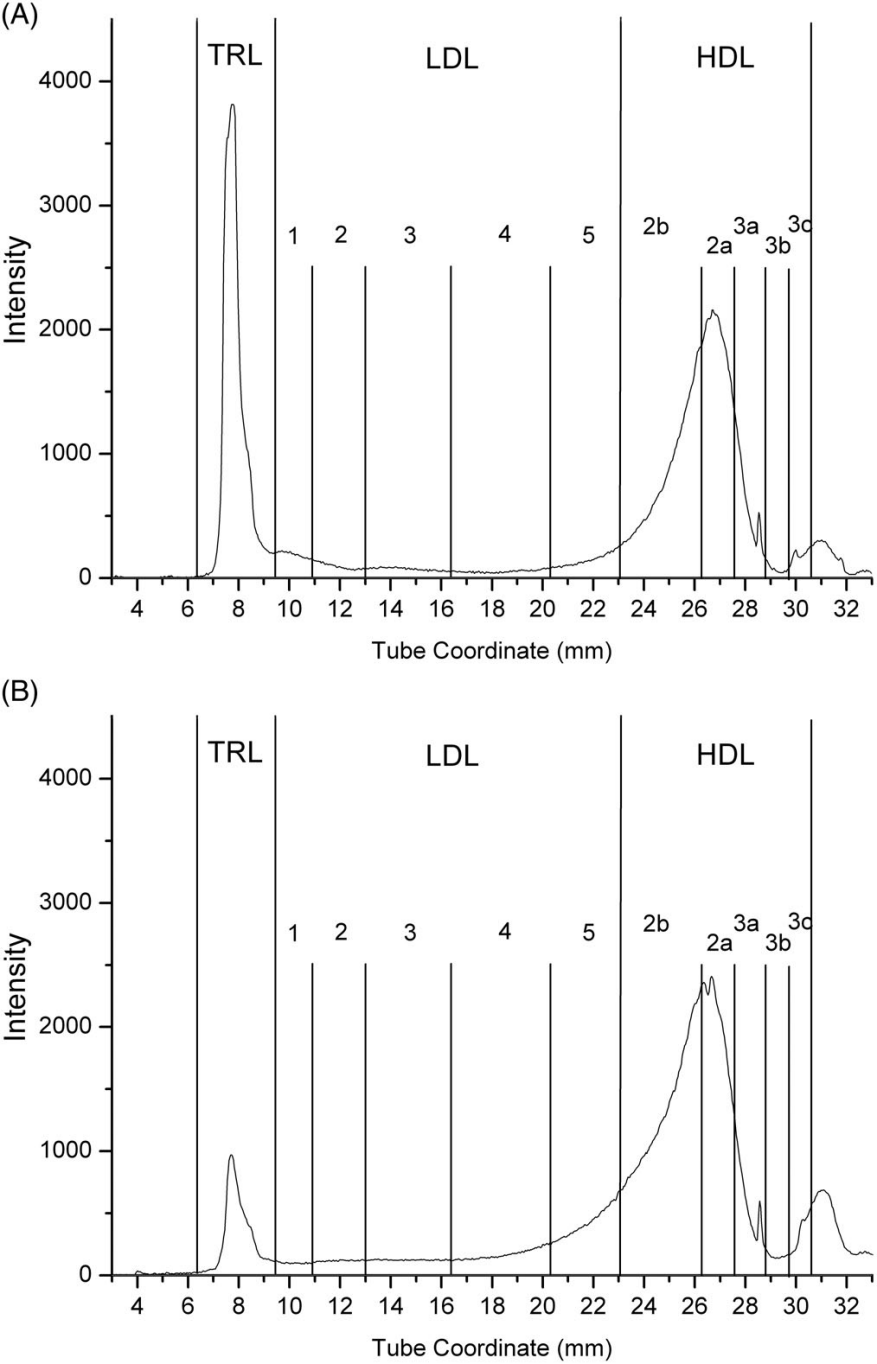
Lipoprotein density profiles from a representative dog in group 1. A, Lipoprotein profile of the dog before the diet change. B, Lipoprotein profile of the same dog 8 weeks after the diet change. This dog had normal serum triglyceride and cholesterol concentrations at that point. Note the dramatic decrease in TRL. A decrease in LDL_1_ and increases in HDL_2b_ and HDL_2a_ are also evident. HDL, high‐density lipoproteins; LDL, low‐density lipoproteins; TRL, triglyceride‐rich lipoproteins

The most important changes in the lipoprotein density distribution of dogs in group 1 in response to diet change were decreases in TRL and LDL_1_ and, to a lesser degree, increases in LDL_4_ and HDL_3c_ (Table 6). Figure 4A,B shows the lipoprotein density profiles from a representative dog in group 1 before and after the diet change.

**TABLE 6:** Mean integrated intensities of the regions of the ultracentrifugation tubes corresponding to 11 distinct density lipoprotein fractions based on density characteristics

Lipoprotein fraction	TRL	LDL1	LDL2	LDL3	LDL4	LDL5	HDL_2b_	HDL_2a_	HDL_3a_	HDL_3b_	HDL_3c_
Before diet change	78 916.93	8748.09	10 192.46	14 490.79	17 298.70	37 863.23	150 732.70	54 421.19	10 513.79	6287.46	11 422.09
After diet change	42 209.95	5574.04	8865.49	15 168.97	21 371.25	41 668.00	146 682.90	55 765.43	12 293.88	7199.44	13 712.91
Direction of change from sample 2 to 3	↓	↓	↓	↑	↑	↑	↓	↑	↑	↑	↑
% change	47	36	13				3				
% change				4	19	9		2	14	13	17

*Note*: Mean integrated intensities before and after diet change as well as the percent of changes are displayed.

Abbreviations: HDL, high‐density lipoproteins; LDL, low‐density lipoproteins; TRL, triglyceride‐rich lipoproteins.

## 4 DISCUSSION

Our study evaluates the effect of a commercially available low‐fat diet on serum lipid concentrations and lipoprotein profiles in MS with hypertriglyceridemia. The results of this study suggest that the study diet was effective in significantly reducing both serum triglyceride and cholesterol concentrations in the majority of hyperlipidemic MS within 2 months of initiation of the study diet. The study diet was effective in lowering and sustaining serum triglyceride concentrations to values below the >500 mg/dL threshold considered to be associated with risk for disease, and in some cases it even led to normalization of serum triglyceride concentrations. The study diet was also effective in reducing serum cholesterol concentrations, but this reduction was less impressive than for serum triglycerides and possibly not as reliably sustained at 12 weeks. In addition, the lipoprotein profiles of hyperlipidemic MS changed significantly within 2 months of initiation of the study diet with more than half of the dogs classified as normal based on SIR analysis of their lipoprotein profiles. Both TRLs and LDL_1_ were the main lipoprotein density fractions affected by low‐fat diet consumption. These 2 fractions decreased while the nominal LDL_4_ and HDL_3c_ density fractions increased in response to low‐fat diet feeding.

Two cut‐off values of serum triglyceride concentrations were used to test the efficacy of the study diet in our study. The reason for this was that the degree of hypertriglyceridemia seems to play an important role in the pathogenesis of most pathologic conditions associated with hypertriglyceridemia.^1^ Mild increases in serum triglyceride concentrations might not be sufficient to induce these disease conditions. For example, in 1 study, serum concentrations of serum triglycerides above approximately 850 mg/dL were found to be associated with an increased risk for an increased Spec cPL concentration in MS, suggesting an increased risk for pancreatitis.^9^ Although evidence‐based recommendations are lacking, in clinical practice, a typical goal is to reduce serum triglyceride concentrations to below 500 mg/dL.^1^ Using either cut‐off value for serum triglyceride concentrations, the study diet was effective in significantly reducing serum triglyceride concentrations in the majority of dogs and only 1 dog had a serum triglyceride >500 mg/dL following 8 weeks of low‐fat diet consumption.

Two samples were collected before the diet change from each dog in group 1. This was done to ensure that dogs had persistent hypertriglyceridemia before the diet change and to determine the variability of serum triglyceride and cholesterol concentrations over time. In a sense, each dog served as its own control in this study. Due to ethical concerns, no actual control group was possible to include in this part of the study because it would have been considered unethical to not provide any means to control hypertriglyceridemia in dogs with confirmed hypertriglyceridemia. Similarly, 2 samples were collected after the diet change, in order to ensure that any reduction in serum triglyceride and cholesterol concentrations was consistent. There was no significant difference in serum triglyceride or cholesterol concentrations between samples 1 and 2 (ie, before the diet change) or between samples 3 and 4 (ie, after the diet change). The fact that significant differences were detected only when serum triglyceride and cholesterol concentrations were compared between time‐points before and after the diet change clearly suggests that this was due to an effect of the diet.

An interesting and unexpected finding of our study was the great variation of serum triglyceride concentrations over time within individual dogs and while consuming the same diet. Figure 1A clearly illustrates this variability as, for example, 1 of the dogs had a serum triglyceride concentration of 300 mg/dL for sample 1 and almost 1400 mg/dL in sample 2. Variation persisted following low‐fat diet consumption as shown by values for samples 3 and 4; however, this variation was not as prominent and was primarily driven by 2 individuals. As mentioned above, there was no statistically significant difference between sample 1 and sample 2 or between sample 3 and sample 4, but there was considerable variation within individual dogs. A plausible explanation is that, because the diet was not controlled during the first 2 sample collections, food of a different fat content might have been consumed before each of the blood collections although the prolonged fasting of these dogs (>12 hours) should theoretically have accounted for such differences. Alternatively, this variation might be normal for this condition, as no long‐term studies have evaluated the fluctuation of serum triglyceride concentration over time within individual MS with hyperlipidemia. Similar variation was observed in another study in MS with pancreatitis.^10^ Although the reason for this variability is currently unknown, it might be suggested that at least 2 samples on different time‐points should be collected during the diagnostic evaluation MS for hypertriglyceridemia to ensure that an accurate picture of the severity of hypertriglyceridemia is obtained.

Another observation was that, although more than 50% of the dogs were classified as normal after the diet change based on their lipoprotein profiles, a considerable percentage (44%) of dogs was still classified as hyperlipidemic based on their lipoprotein profiles. This is in agreement with the observation that despite significant reduction in serum triglyceride concentrations, values were not completely normalized in many dogs (Figure 1). It is not known why the dogs responded to the diet change to different degrees. One possible explanation is that some dogs might need more time than 2 to 3 months to fully respond to the diet. Although there are no studies convincingly showing how much time is required for normalization of serum triglyceride concentrations after feeding a low‐fat diet, clinical experience suggests that 2 to 3 months is usually sufficient. Another explanation might be that the common phenotype of hypertriglyceridemia in MS might have different underlying genetic and biochemical bases, and therefore, different treatment requirements. This possibility deserves further study. Finally, it is possible that some dogs had unidentified conditions that cause secondary hypertriglyceridemia (eg, low‐grade chronic pancreatitis, hyperadrenocorticism) and that these dogs did not respond because the primary cause was still present. However, it has not been proven that very mild pancreatitis or hyperadrenocorticism that cause no clinical signs can cause lipid abnormalities.

It should be noted that while all MS in group 1 were neutered, in group 2 some of the dogs (15/28) were not. Although there are no studies that show that neutering significantly changes lipoprotein profiles in MS, it is possible that some of the differences in the lipoprotein profiles between groups 1 and 2 might have been affected by the difference of the sexual status. In addition, statistical analysis after removing intact dogs did not show any differences in the results. Differences in the lipoprotein profiles found in group 1 dogs before and after diet intervention were made without consideration or comparison to group 2 dogs and so are independent of sexual status.

It is of note that the baseline lipoprotein profiles (ie, before the diet change) of the dogs that eventually responded to the diet change were distinctly different from the ones that did not fully respond to the diet. This further suggests that hypertriglyceridemia in MS is a clinically diverse condition, which might have different underlying genetic and biochemical bases, and that certain phenotypes of the disease might not be as responsive to dietary management with low‐fat diets compared to others. Data from our study but also from older studies suggest that MS often have different biochemical abnormalities associated with hyperlipidemia. For example, in 1 study it was shown that some hyperlipidemic MS have hyperchylomicronemia while others do not.^6^ Similarly, some MS with hypertriglyceridemia also have hypercholesterolemia while others do not.^4^, ^6^ Chylomicron formation depends upon dietary fat consumption and hyperchylomicronemia is expected to respond to low‐fat diets.^17^ In contrast, increases in VLDL, which are also triglyceride‐rich and isolated in the TRL fraction with our methodology, are not necessarily responsive to low‐fat diets because VLDLs are produced through the endogenous pathway and contain triglycerides derived from de novo lipogenesis.^17^ Our study supports the notion that lipoprotein profile analysis might be useful in the clinical evaluation of hyperlipidemic dogs because it provides important additional information to serum triglyceride and cholesterol concentration. In particular, an improved ability to predict responsiveness to a low‐fat diet intervention might affect clinical decision making with regards to management of dogs with hyperlipidemia. It needs to be noted, however, that unidentified causes of secondary hypertriglyceridemia might have accounted for these differences in lipoprotein profiles, although this is considered unlikely.

The most prominent changes in the lipoprotein density profile involved decreases in TRLs and LDL_1_. This is consistent with significant reductions in serum triglyceride and cholesterol concentrations. Triglyceride‐rich lipoproteins include VLDLs and chylomicrons, which are responsible for transporting serum triglycerides. Both serum triglyceride and cholesterol concentrations decreased significantly after the diet change and, therefore, lipoproteins that transfer large amounts of triglycerides or cholesterol were also decreased. These decreases are reflected in the lipoprotein fingerprint with the reductions in TRLs and LDL_1_. Some other lipoprotein fractions (ie, LDL_4_ and HDL_3c_) increased after diet change. The exact content and function of canine lipoprotein fractions has not yet been investigated and thus, a detailed explanation of each 1 of the changes observed in the lipoprotein density distribution in response to diet change is not possible at this point.

## 5 CONCLUSION

Our study evaluated the effect of a commercially available low‐fat diet on serum lipid concentrations and lipoprotein profiles in MS with hyperlipidemia. The study diet was effective in significantly reducing serum triglyceride and cholesterol concentrations within 2 months. The lipoprotein profiles of most hyperlipidemic dogs were shifted toward those of nonhyperlipidemic dogs within 2 months. A subgroup of dogs did not fully respond to the diet change as indicated mainly by their serum triglyceride concentrations and lipoprotein profiles. The reason for this finding is unknown, but differences in the pathogenetic basis of hypertriglyceridemia among MS might be involved. Unidentified causes of secondary hypertriglyceridemia might also be involved. Given the fact that the dogs enrolled in the present study had naturally occurring hyperlipidemia, the study diet should be expected to be beneficial in clinical practice. It is unknown whether other low‐fat diets would have the same effect on the management of hypertriglyceridemia in MS. Further studies are needed and underway to evaluate the genetic basis and possible genetic differences of hypertriglyceridemia in this and other breeds.

## CONFLICT OF INTEREST DECLARATION

Authors declare no conflict of interest.

## OFF‐LABEL ANTIMICROBIAL DECLARATION

Authors declare no off‐label use of antimicrobials.

## INSTITUTIONAL ANIMAL CARE AND USE COMMITTEE (IACUC) OR OTHER APPROVAL DECLARATION

The study protocol was reviewed and approved by the Clinical Research Review Committee at Texas A&M University (TAMU‐CRRC#2008‐37).

## HUMAN ETHICS APPROVAL DECLARATION

Authors declare human ethics approval was not needed for this study.
